# Early-Life Low Lead Levels and Academic Achievement in Childhood and Adolescence

**DOI:** 10.1001/jamanetworkopen.2025.12796

**Published:** 2025-05-28

**Authors:** George L. Wehby

**Affiliations:** 1 Department of Health Management and Policy, University of Iowa, Iowa City; 2 Department of Economics, University of Iowa, Iowa City; 3 National Bureau of Economic Research, Cambridge, Massachusetts

## Abstract

**Importance:**

In 2021, the Centers for Disease Control and Prevention lowered the blood lead reference values to 3.5 μg/dL or higher. More evidence is needed on the association between lead changes below 3.5 μg/dL and academic achievement.

**Objective:**

To examine the association of a 1-unit change in early childhood blood lead levels below 3.5 μg/dL vs at 3.5 μg/dL or higher with academic achievement.

**Design, Setting, and Participants:**

This cohort study linked birth certificates of children born in Iowa from 1989 to 2010 to their math and reading test scores for grades 2 through 11 and to their early childhood blood lead testing data. Analyses were completed between May 2024 and March 2025.

**Exposures:**

Early life blood lead levels below 3.5 μg/dL and at 3.5 μg/dL or higher.

**Main Outcome and Measure:**

The primary outcome was national percentile rank (NPR) scores on math and reading school tests. Regression models estimated the association between lead levels and math and reading scores, controlling for sociodemographic, child and maternal health, and school characteristics.

**Results:**

The analytical sample included up to 305 256 children and 1 782 873 child-grade observations. Within the analytical sample, 51.0% were male, 41.1% were first-born children, and 43.9% were born to mothers with an educational level of high school or less. Sociodemographic and child and maternal health characteristics were similar between the analytical sample and the birth population. The mean (SD) child age at lead testing was 1.9 (1.5) years. Among these children, 37.7% had lead values below 3.5 μg/dL. A 1-unit increase in lead levels below 3.5 μg/dL was associated with lower NPR scores in math by −0.47 (95% CI, −0.65 to −0.30) and in reading by −0.38 (95% CI, −0.56 to −0.20). For lead levels at 3.5 μg/dL or higher, a 1-unit increase in lead levels was associated with lower NPR scores in math by −0.52 (95% CI, −0.58 to −0.47) and in reading by −0.56 (95% CI, −0.62 to −0.51). These score declines were persistent across grades 2 through 11.

**Conclusions and Relevance:**

In this cohort study assessing early life low lead level and children’s and adolescents’ academic achievement, a 1-unit increase in lead levels in the range currently considered low for further interventions was associated with worse academic performance throughout school grades comparable to that for lead levels in the range recommended for additional interventions. These findings support the need to reconsider and potentially lower current blood lead reference values for recommending further interventions.

## Key Points

**Question:**

Is there an association of a 1-unit increase in early childhood blood lead levels below 3.5 μg/dL with academic achievement compared with 3.5 μg/dL or higher?

**Findings:**

In this cohort study of up to 305 256 children and 1 782 873 child-grade observations, a 1-unit increase in blood lead levels below 3.5 μg/dL was associated with worse math and reading scores in grades 2 through 11, comparable to those associated with blood lead levels of 3.5 μg/dL or higher.

**Meaning:**

These findings suggest that it is important to reconsider and potentially lower the current blood lead reference values (≥3.5 μg/dL) for recommending further interventions.

## Teaser

This cohort study linking birth certificates, blood lead levels, and reading and math test scores investigates whether there is an association between the current lead reference values vs lower lead levels for children and academic achievement in grades 2 through 11.

## Introduction

Children’s exposure to lead has long been recognized as harmful to their health and neurodevelopment. Lead biologically disrupts and damages brain and neurological development. Lead exposure has been associated with long-term adverse cognitive, mental, physical, and social outcomes.^1,2,3,4^ Globally, nearly 5.5 million cardiovascular-related deaths among adults and 765 million lost IQ points among children below 5 years of age in 2019 were associated with lead exposure.^5^

National and international public health authoritative bodies, such as the Centers for Disease Control and Prevention (CDC) and the World Health Organization, acknowledge that there are no safe levels of lead.^6,7^ Such organizations provide guidelines based on thresholds for blood lead levels that are considered high enough to warrant retesting, assessment of lead exposure sources, parent education about lead exposure, and in cases of high lead levels, treatment of children for these levels.^8^ During the past few decades, these thresholds have become lower as the mean lead blood level and the proportion of children with high blood levels steadily declined, owing to a range of health and housing policies aimed at eliminating or reducing lead exposure from homes, canned food, water, gasoline, and other sources. Because of these policies and reduced exposure to lead, the mean lead blood level among children aged 1 to 5 years in the US declined from 15.2 to 0.83 μg/dL between 1976 through 1989 and 2011 through 2016 based on samples from the National Health and Nutrition Examination Survey (NHANES).^9^ Nonetheless, lead continues to affect many children and adults worldwide. In 2015, over half of the US population was estimated to have been exposed to high lead levels in early childhood.^10^

The CDC has historically issued guidelines for blood lead levels. As stated by the CDC, these are not meant as health or toxicity standards but represent the population-based levels for the 2.5% of children in NHANES with the highest blood lead values.^11^ These thresholds are referred to as blood lead reference values. In 2021, the CDC lowered the threshold from 5 μg/dL or higher, which was in effect from 2012 to 2021, to 3.5 μg/dL or higher; these thresholds represent the 97.5th percentiles in 2015 through 2018 and 2007 through 2010 samples, respectively.^11^ Before 2012, the CDC had 10 μg/dL or higher as a level of concern.^11^

The CDC and other authoritative public health agencies acknowledge that low blood lead levels can still be harmful for child development. However, current thresholds (and those before them) are also meant to guide government and public health (federal, state, and local) processes and resources for further interventions. For lead levels greater than or equal to 3.5 μg/dL, recommendations include additional management and intervention steps for reporting the test, assessing and investigating environmental exposure history, diet checks and assessments especially for calcium or iron deficiency, evaluating development, considering referral to additional services and specialists, and above certain thresholds (>45 μg/dL) gastrointestinal tract decontamination.^8,12^

To better inform the thresholds for surveillance and interventions, it is important to understand how blood lead levels currently considered to be in low ranges affect children’s development. Some evidence comes from population-based linkages of lead testing and academic achievement data. One study that examined blood lead levels in single-unit ranges up to 10 μg/dL and children’s reading and math scores between grades 3 and 8 in 2000 through 2012 in North Carolina found lower scores for children with blood levels above 1 μg/dL that persist over grades.^13^ An earlier study that found associations between lead tests and fourth grade reading and math tests in 7 counties in North Carolina also reported lower grades with higher lead levels, including lead values of 2 or 3 μg/dL relative to 1 μg/dL or lower.^14^ Another study that assessed lead levels and third grade school tests for children born between 1997 and 2005 in Rhode Island, with the majority (83%) having mean lead values below 5 μg/dL, also found lower reading and math scores with an increase in lead levels.^15^ One study that found associations between lead level tests and third grade school tests for children born in Chicago between 1994 and 1998 also reported higher risk of failing on reading or math with a 5-μg/dL increase in lead levels.^16^ In addition to these population-based associations of lead levels and school test results, a study of children aged 6 to 16 years in NHANES III from 1988 to 1994 also reported declines in arithmetic and reading tests associated with an increase in lead levels below 5 μg/dL.^17^ Other studies show that cognitive declines are observed earlier than school age based on IQ or mental and psychomotor development outcomes.^18,19^

The present study examines the association between a 1-unit change in early childhood blood lead levels for what is currently considered a low range per CDC standards (ie, <3.5 μg/dL) and children’s academic achievement measured by standardized math and reading tests across grades 2 through 11. The associations were estimated both pooling by grade and separately for each grade from 2 through 11. To compare with lead changes in the high range, the study also evaluated the association with a 1-unit change in blood lead levels at 3.5 μg/dL or higher. The study links birth certificates, blood lead tests, and academic tests for a large sample of children from an entire state (Iowa). Birth certificates provide data on sociodemographic and child and maternal health covariates. The school assessments are highly reliable with national norms and are regularly administered to virtually all students in public and private schools in Iowa.^20^ Previous studies have found associations between child health indicators and those school assessments that support their validity for studying child health risks and academic achievement.^21,22,23,24,25^ Reporting blood lead test results to the Iowa Department of Health and Human Services (Iowa HHS, formerly the Iowa Department of Public Health) is mandatory.^26^ All lead tests were completed for children aged 0 to 7 years, with more than half completed by 1 year of age (58.9%) and almost all by 5 years of age (97.6%). Beginning in 2008, children in Iowa were required to have had at least 1 lead test before entering kindergarten. For children with lead values below 10 μg/dL, the general guideline is to continue routine testing as recommended.^27^ The present study assesses whether there is an association between changes in lead levels for what is currently considered to be a low lead range (<3.5 μg/dL) and children’s subsequent school achievement.

## Methods

### Data and Sample

This cohort study linked the following 3 population-based data sources: (1) Iowa birth certificates from 1989 to 2010 from Iowa HHS; (2) standardized tests of academic achievement (Iowa Tests of Basic Skills, Iowa Tests of Educational Development, and Iowa Assessments) from the Iowa Testing Programs available through 2017-2018; and (3) lead testing data from 1990 through 2017 from Iowa HHS. Linkage was accomplished using the child’s name and birth date independently of the researchers who only had access to deidentified data. Data linkages and the sensitivity of the results to the matching rates were approved by the University of Iowa institutional review board, which also waived informed consent because deidentified data were used. This study follows the Strengthening the Reporting of Observational Studies in Epidemiology (STROBE) reporting guidelines for cohort studies.

eFigure 1 in Supplement 1 shows the sample construction flow. The analytical sample was limited to singleton births with birth weight between 500 and 6000 g and gestational age between 20 and 44 weeks. Of all singleton births in Iowa in 1989 through 2010, 77.5% were matched to school tests. eFigure 2 in Supplement 1 shows the matching rates between birth certificates and school tests by birth year, which were approximately 80% for births in 1989 through 2008. The matching rates were lower in 2009 and 2010 (56.4% and 14.9%) because of limiting the sample to second grade or higher grades, the lower testing rate at early grades, and school test data availability through school year 2017-2018. Of the children with matched birth certificate and school test data, 51.9% were also matched to lead testing data. eFigure 3 in Supplement 1 shows those matching rates by birth year; matching rates steadily increased from 12.7% in 1989 to over 50% in 2000 and approximately 80% from 2004 through 2010 because of the increase in lead testing over time. A sensitivity analysis was conducted to evaluate the outcome of matching rates on the estimates. It is important that the matched sample be similar to the population of singleton births in Iowa on sociodemographic and child and maternal health characteristics.

### Statistical Analysis

Study outcomes were the child’s math and reading scores in grades 2 through 11 measured as the national percentile rank (NPR), which is the child’s percentile score relative to a national sample. Blood lead levels were based on the geometric mean of all blood tests the child may have undergone (50.7% of children in the analytical sample had 1 lead test, with the remainder having more than 1 test).

The following regression model estimated the association of a 1-unit increase in lead levels with school test scores separately for children with lead levels below 3.5 μg/dL (to convert to μmol/L, multiply by 0.0483) and those with lead levels of 3.5 μg/dL or higher: *Test Score_i_* = β_0_ + β_1_ *Lead_i_* + X_i_ λ + *e_i_*. For child *i* with a school test score in a given grade, the *Test Score* is on either math or reading, and *Lead* is blood lead level (geometric mean if multiple tests). *X* is a vector of child, maternal, and school characteristics. The child’s covariates included a 0 or 1 indicator for month and year of birth, sex, gestational age (0 or 1 indicator for each gestational age in weeks), birth weight (0 or 1 indicator for birth weight deciles), 0 or 1 indicator for congenital anomalies, and 0 or 1 indicator for first year of lead testing. By including the 0 or 1 indicators for birth year and first year of lead test, the model accounted for age at lead testing. Maternal covariates included the following self-reported sociodemographic factors: age based on delivery date and reported maternal date of birth (0 or 1 indicator for each age in years), marital status, educational level, and race and ethnicity (from birth certificate data), in addition to a 0 or 1 indicator for smoking during pregnancy. Race and ethnicity were provided in the data in categories of Asian or Pacific Islander, Black, Native American, White, or unknown. Maternal race was included in the model as a 2-category indicator of White vs non-White to have minimum sufficient frequencies for this variable. Additional maternal covariates from hospital records included the number of prior live births (at child’s birth), number of prenatal visits, 0 or 1 indicator for cesarean delivery, 0 or 1 indicator for labor induction, and 0 or 1 indicator for perinatal complications. School covariates included 0 or 1 indicators for school districts and for year, grade (when pooling by grade), and semester at the time of school test. The regression was estimated by ordinary least squares, both pooled and separately by grade; standard errors were clustered at the child level when pooling by grade.

Additional models were estimated, including 4 sensitivity checks and a heterogeneity analysis by child’s sex. In earlier years and in some testing laboratories, lead values of 5 μg/dL or less were reported as 5 μg/dL. Therefore, the model was reestimated excluding observations with 5 μg/dL. Detection limits in later years were much less than 5 μg/dL and as low as 1 μg/dL. Another analysis limited the sample to births years 2000 through 2009, which had a higher matching rate (58.5% of singleton births matched to school test and lead data) than the total sample. A third analysis added school district time-specific trends (interactions between the school district and year of birth 0 or 1 indicator) as covariates to allow for local changes in academic outcomes and lead levels over time. This analysis leveraged differences within children in the same school district while allowing for districts to have their own time trends. Another sensitivity check excluded birth weight deciles and maternal smoking as covariates since prior research suggests that maternal low lead levels are associated with lower birth weight and that maternal smoking is associated with higher children’s lead levels.^28,29^ Lastly, prior research (albeit based on small samples) suggests larger effect sizes among males than females for prenatal lead exposure associated with cognitive outcomes.^30^ Therefore, another analysis in the present study was stratified by child’s sex. These additional analyses were estimated pooling by grade level.

All analyses were conducted with Stata, version 15.1 (StataCorp LLC). Statistical significance was defined as a 95% CI excluding 0. Analyses were completed between May 2024 and March 2025.

## Results

### Sample Description

The total analytical sample with complete data on outcomes and covariates included 304 202 unique children with 1 775 499 child-grade observations for math scores and 305 256 unique children with 1 782 873 child-grade observations for reading scores. In this sample, 49.0% of children were female, 51.0% were male, and 41.1% were first-born children. In addition, 93.2% of children were born to mothers who self-reported being White (6.8% selected one of the other race categories available, including Asian or Pacific Islander, Black, Native American, or unknown, which were combined in the analysis into 1 group) (Table 1). Moreover, 43.9% of children in this analytical sample were born to mothers who had an educational level of high school or less, 30.9% to mothers with some college, and 25.3% to mothers with a college education. The mean (SD) maternal age was 26.7 (5.6) years. The mean (SD) child age at lead testing was 1.9 (1.5) years. Among these children, 37.7% had lead values below 3.5 μg/dL, with a mean (SD) of 2.3 (0.8) μg/dL in that group. Children with values of 3.5 μg/dL or higher had mean (SD) lead values of 5.7 (2.1) μg/dL.

**Table 1.** Comparison of Birth Population and Included Samples on Sociodemographic and Health Variables

Characteristic	Participant, No. (%)^a^		
	Population	Sample	
		Matched	Analytical
Child’s sex			
Female	405 738 (48.8)	155 452 (49.0)	148 926 (49.0)
Male	386 341 (51.2)	162 119 (51.0)	155 276 (51.0)
No. of prior live births			
0	314 002 (39.7)	130 405 (41.1)	125 088 (41.1)
1	262 175 (33.1)	105 701 (33.3)	101 466 (33.4)
2	137 886 (17.4)	53 752 (16.9)	51 529 (16.9)
3	50 452 (6.4)	19 055 (6.0)	18 178 (6.0)
≥4	26 838 (3.4)	8423 (2.7)	7941 (2.6)
Maternal age at child’s birth, mean (SD), y	26.8 (5.6)	26.6 (5.6)	26.7 (5.6)
No. of observations	792 037	317 559	304 202
Maternal race^b^			
White	732 991 (92.8)	294 886 (93.1)	283 538 (93.2)
Non-White	56 843 (7.2)	21 837 (6.9)	20 664 (6.8)
Maternal Hispanic ethnicity			
Yes	42 596 (5.4)	16 298 (5.2)	15 021 (5.0)
No	746 797 (94.6)	300 247 (94.9)	289 181 (95.1)
Maternal marital status at child’s birth			
Married	563 928 (71.2)	214 179 (67.5)	205 875 (67.7)
Not married	227 976 (28.8)	103 320 (32.5)	98 327 (32.3)
Maternal educational level at child’s birth			
Less than high school	111 691 (14.2)	44 915 (14.2)	42 498 (14.0)
High school	235 711 (30.0)	95 053 (30.1)	90 872 (29.9)
Some college	235 823 (30.0)	96 881 (30.7)	93 883 (30.9)
College graduate	203 527 (25.9)	78 969 (25.0)	76 949 (25.3)
Birth weight, mean (SD), g	3419 (549)	3408 (542)	3410 (541)
No. of observations	791 025	317 492	304 202
Gestational age, mean (SD), wk	39.0 (1.9)	38.9 (1.8)	38.9 (1.8)
No. of observations	789 239	316 881	304 202
Prenatal visits, mean (SD)	11.8 (3.3)	12.0 (3.1)	12.0 (3.1)
No. of observations	776 900	313 036	304 202
Maternal smoking during pregnancy			
Yes	150 134 (19.0)	62 679 (19.7)	59 508 (19.6)
No	641 581 (81.0)	254 766 (80.3)	244 694 (80.4)
Child had congenital anomalies			
Yes	2536 (0.3)	675 (0.2)	641 (0.2)
No	789 545 (99.7)	316 896 (99.8)	303 561 (99.8)
Mother had health complications			
Yes	46 216 (5.8)	19 952 (6.3)	18 968 (6.2)
No	745 865 (94.2)	297 619 (93.7)	285 234 (93.8)
Labor induction			
Yes	173 908 (22.0)	78 824 (24.9)	75 900 (25.0)
No	615 867 (78.0)	238 253 (76.2)	228 302 (75.1)
Cesarean delivery			
Yes	175 653 (22.2)	75 508 (23.8)	72 644 (23.9)
No	614 587 (77.8)	241 794 (76.2)	231 558 (76.1)

^a^ Descriptive statistics are based on unique children (not child-grade observations). The population includes 792 081 singleton-born children born in Iowa from 1989 to 2010. The matched sample includes 317 571 singleton-born children matched between the 3 datasets (birth certificates, school tests, and lead tests). The analytical sample of 304 202 children is the subset of the matched sample with data on math scores and complete data on all covariates. The equivalent analytical sample for reading scores includes 305 256 observations. Frequencies may not sum to 100% due to rounding. The sum of sample counts across categories of a variable may not add up to the total count for the population or matched sample due to missing data.

^b^ Maternal race was included as a 2-category indicator of White vs non-White to have minimum sufficient frequencies for this variable. The non-White category included Asian or Pacific Islander, Black, Native American, and unknown.

Table 1 presents a comparison of the sociodemographic and child and maternal health characteristics of the population of singleton births in Iowa in the study years, the matched sample, and analytical sample with complete data. Overall, these characteristics were similar between the analytical and the birth population, and differences were generally small (eg, 67.7% of the analytical sample were married relative to 71.2% of the total population). The mean (SD) NPR math and reading scores in the analytical sample were 59.9 (27.2) and 59.2 (27.7), respectively.

### Association of Lead Levels With Academic Achievement

Table 2 reports the estimated associations between a 1-unit increase in mean blood lead levels and math and reading scores from the regression pooling by grade. Separate estimates are provided for lead values below 3.5 μg/dL and for 3.5 μg/dL or higher. A 1-unit increase in lead levels below 3.5 μg/dL was associated with declines in NPR math and reading scores by −0.47 (95% CI, −0.65 to −0.30) and −0.38 (95% CI, −0.56 to −0.20), respectively. For lead levels of 3.5 μg/dL and higher, a 1-unit increase in lead levels was associated with declines in NPR scores for math and reading by −0.52 (95% CI, −0.58 to −0.47) and −0.56 (95% CI, −0.62 to −0.51), respectively.

**Table 2.** Association Between a 1-Unit Increase in Lead Levels and Math and Reading Scores, for Lead Levels Below 3.5 μg/dL and for 3.5 μg/dL or Higher

Measure	Lead level			
	<3.5 μg/dL		≥3.5 μg/dL	
	NPR for math	NPR for reading	NPR for math	NPR for reading
Estimate (95% CI)	−0.47 (−0.65 to −0.30)	−0.38 (−0.56 to −0.20)	−0.52 (−0.58 to −0.47)	−0.56 (−0.62 to −0.51)
No. of child-grade observations	562 447	563 380	1 213 052	1 219 493

Abbreviation: NPR, national percentile rank.

SI unit conversion: To convert lead to μmol/L, multiply by 0.0483.

**Figure 1.**
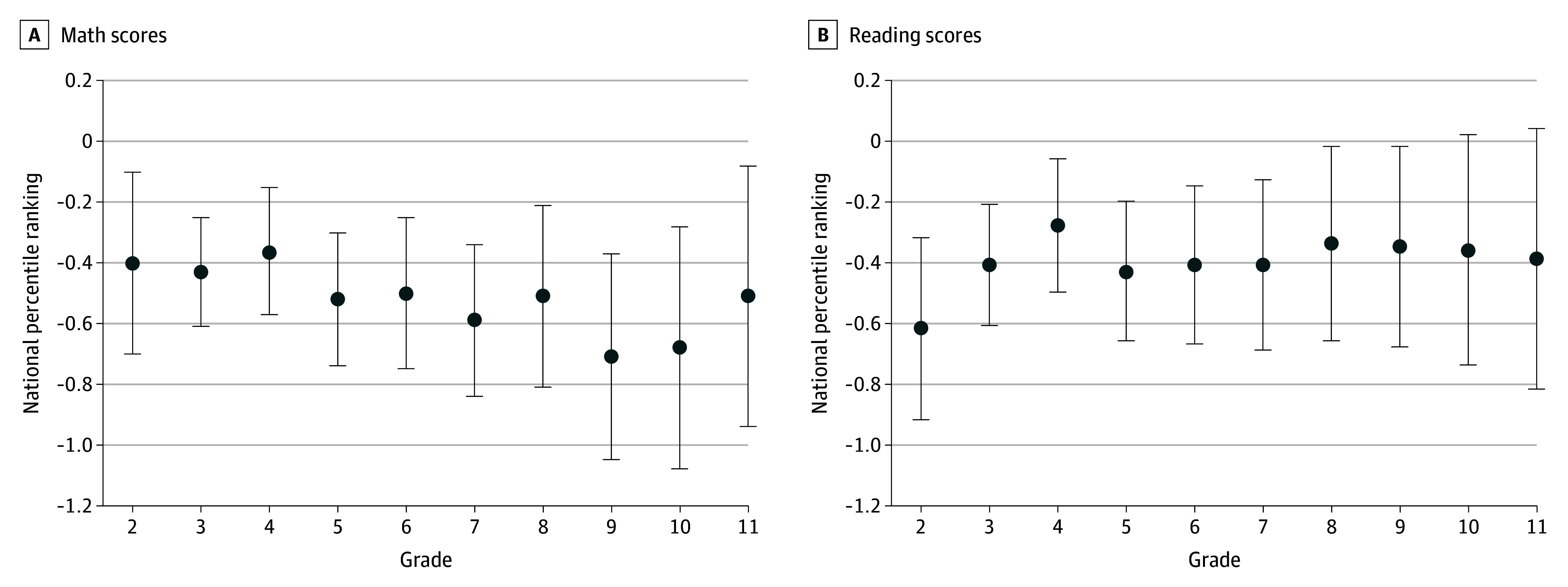
Association of a 1-Unit Increase in Lead Levels and Math and Reading Scores by Grade for Lead Levels Below 3.5 μg/dL Estimates (dots) represent the association of a 1-unit increase in lead levels with math and reading scores estimated separately for each grade; bars, 95% CIs.

Figure 1 reports the associations between a 1-unit increase in blood lead levels and math and reading scores separately for each grade from grade 2 through grade 11 for children with lead ranges below 3.5 μg/dL (detailed estimates in eTables 1 and 2 in Supplement 1). There was a decline in NPR math scores in each grade ranging between −0.36 (95% CI, −0.57 to −0.16) per 1-unit increase in lead values for grade 4 and −0.71 (95% CI, −1.05 to −0.37) per 1-unit increase in lead values for grade 9. Similarly, there were declines in NPR reading scores in each grade ranging between −0.28 (95% CI, −0.50 to −0.066) per 1-unit increase in lead values for grade 4 and −0.62 (95% CI, −0.92 to −0.33) per 1-unit increase in lead values for grade 2. NPR reading score declines in grades 10 and 11 were not statistically significant.

**Figure 2.**
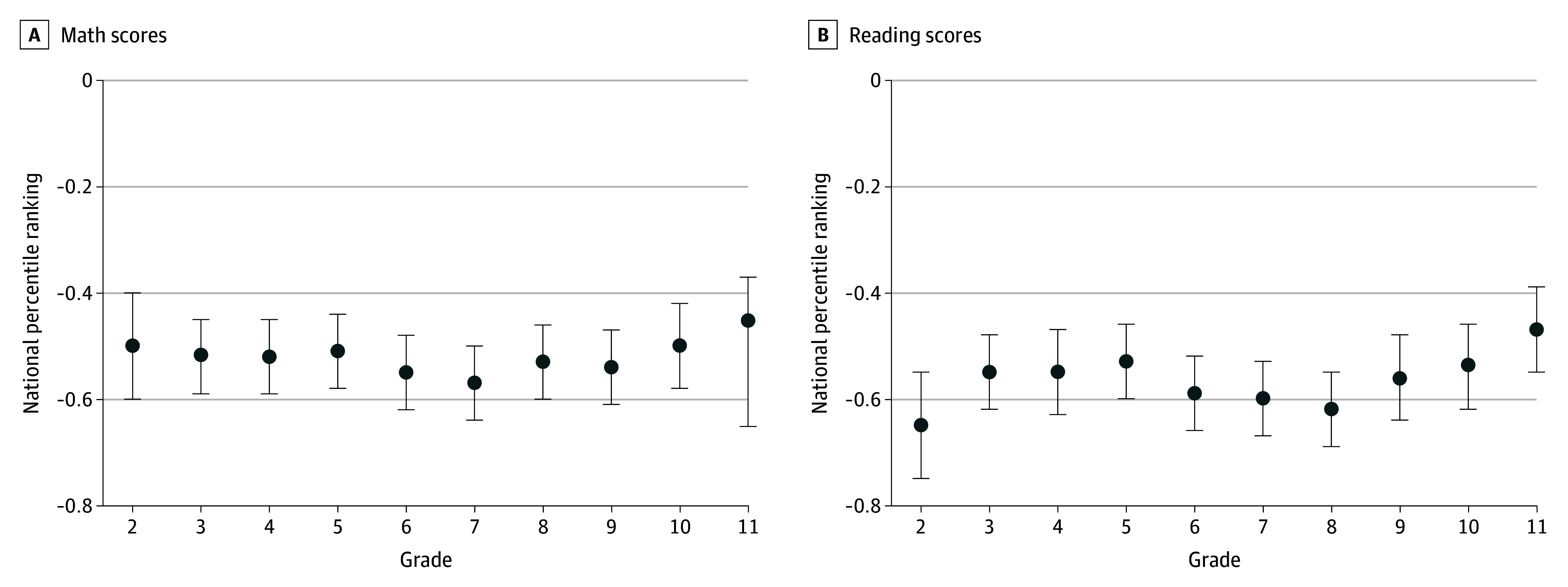
Association of a 1-Unit Increase in Lead Levels and Math and Reading Scores by Grade for Lead Levels 3.5 μg/dL or Higher Estimates (dots) represent the association of a 1-unit increase in lead levels with math and reading scores estimated separately for each grade; bars, 95% CIs.

Figure 2 reports the grade by grade estimates for associations of 1-unit increase in lead levels with math and reading scores for children with lead levels of 3.5 μg/dL or higher (detailed estimates in eTables 3 and 4 in Supplement 1). These estimates show comparable declines by grade level that are within ranges of those for lead levels below 3.5 μg/dL.

### Additional Analyses

eTable 5 in Supplement 1 reports the estimates of lead level associations with school test scores excluding 5 μg/dL lead values for children with only 1 blood lead test or when calculating the lead value means for children with multiple tests. When excluding 5 μg/dL lead values, the estimates were slightly smaller for lead levels below 3.5 μg/dL, with a 1-unit increase in lead level associated with lower NPR math scores by −0.37 (95% CI, −0.55 to −0.20) and lower NPR reading scores by −0.30 (95% CI, −0.48 to −0.12). These estimates were similar to those for 3.5 μg/dL or higher lead levels, with a decline of 0.31 (95% CI, −0.37 to −0.25) in math and reading scores per 1 unit-increase in lead levels.

eTables 6 and 7 in Supplement 1 report estimates from the sensitivity analyses limiting the sample to birth years from 2000 to 2009 and adding school district–specific time trends, respectively. These estimates were similar to the main estimates (Table 2). eTable 8 in Supplement 1 reports estimates from the model excluding birth weight and maternal smoking as covariates; these estimates were also comparable to the main results. Lastly, eTable 9 in Supplement 1 reports the estimates separately for males and females. Declines in math and reading scores with higher lead levels were slightly more pronounced among females than males for lead values below 3.5 μg/dL and were similar between males and females for lead values of 3.5 μg/dL or higher.

## Discussion

This cohort study investigated the association of blood lead levels in early childhood with subsequent school achievement in ranges below (<3.5 μg/dL) and above (≥3.5 μg/dL) current CDC thresholds for high lead levels. Linking population-based data from Iowa on birth certificates, lead tests, and math and reading test scores for grades 2 through 11, this study found that a 1-unit increase in lead levels in the range currently considered low for individual-level interventions (<3.5 μg/dL) was associated with worse academic performance throughout school grades. The declines in achievement per 1 additional unit of lead levels were close to those observed in the range currently recommended for intervention (≥3.5 μg/dL). Moreover, the declines in school tests associated with lead levels were generally steady across all grades, highlighting the persistence and permanency of the academic deficits associated with lead exposure.

The study adds evidence on the association between low lead levels and children’s academic achievement through high school, and the findings are consistent with prior research, including a study examining grade by grade changes through middle school (grade 8).^13^ The present work provides further evidence to support that there are no safe levels of lead and that there is a need to continue to reduce or eliminate lead exposure. Also, this evidence along with previous studies suggests a need to reconsider the lead reference values for recommending individual-level interventions to reduce long-term developmental risks.

### Strengths and Limitations

One study strength was the population-based and large sample that included 40% of all singleton births in Iowa across the study years (58.5% when limiting the sample to birth years 2000 through 2009). Moreover, the study sample was representative of the sociodemographic and child and maternal health characteristics of the birth population, with no evidence of systematic differences between the analytical sample and the population. Also, the results were similar when limiting the sample to more recent birth years with higher matching rates. Collectively, these findings support the generalizability of the results from the matched sample to the total population of births in Iowa. Another strength of the study was the detailed child-level data on lead levels in early childhood and on school test scores through most grades from elementary through high school in addition to the grade by grade analyses. The model also accounted for several conceptually relevant child and maternal covariates. Additionally, in one specification, the model allowed for differences in school outcomes across school districts over time, which isolated the variation in lead exposure to within school district changes in lead exposure over time, thus controlling for potential local area confounders.

This study also has limitations. One issue was that several laboratories recorded lead levels considered in older guidelines to not be in the high range at 5 μg/dL. A sensitivity analysis excluding 5 μg/dL values showed overall comparable results. Even though the estimates were slightly smaller, they were comparable between the ranges below 3.5 μg/dL and 3.5 μg/dL or higher. Another limitation was the possibility of residual confounding, which cannot be ruled out. As a study of existing data, covariates were limited to what was available, and some conceptually relevant variables, such as family income or residence quality, were not measured. However, it is reassuring that the study findings aligned well with previous studies with different data sources, settings, and designs. Additionally, the study had no data on any specific lead-related interventions that children may have received to consider their potential effects. Such interventions, however, were not confounders but rather potential effect modifiers, and they were part of the overall association of lead exposure with academic achievement. However, understanding the impacts of interventions to counter their associations with lead in future work is important.

## Conclusions

In this cohort study assessing early life low blood lead level and children’s and adolescents’ academic achievement, a 1-unit increase in lead levels in the range currently considered low for interventions (<3.5 μg/dL) was associated with worse academic performance, close to the achievement decline per 1-unit increase in the range recommended for intervention (≥3.5 μg/dL). The achievement decline was observed across grades in elementary, middle, and high school. Reconsidering and potentially lowering current blood lead reference values for intervention may be needed to better address the associations of low-level lead exposures with cognitive and academic outcomes.

## Notes

Supplement 1. **eFigure 1.** Sample Construction **eFigure 2.** Matching of Birth Certificate and School Test Data Rates by Birth Year **eFigure 3.** Percent of Children with Matched Birth Certificates and School Test Data Also Matched With Lead Testing Data by Birth Year **eTable 1.** Regression Estimates of Association of a 1-Unit (µg/dL) Increase in Lead Levels and Math Scores Within Lead Levels <3.5 µg/dL by Grade **eTable 2.** Regression Estimates of Association of a 1-Unit (µg/dL) Increase in Lead Levels and Reading Scores Within Lead Levels <3.5 µg/dL by Grade **eTable 3.** Regression Estimates of Association of a 1-Unit (µg/dL) Increase in Lead Levels and Math Scores Within Lead Levels ≥3.5 µg/dL by Grade **eTable 4.** Regression Estimates of Association of a 1-Unit (µg/dL) Increase in Lead Levels and Reading Scores Within Lead Levels ≥3.5 µg/dL by Grade **eTable 5.** Association Between 1-Unit (µg/dL) Increase in Lead Levels and Math and Reading Scores Within Lead Levels <3.5 µg/dL and ≥3.5 µg/dL Excluding 5 µg/dL Values From Lead Mean Calculation **eTable 6.** Association Between 1-Unit (µg/dL) Increase in Lead Levels and Math and Reading Scores Within Lead Levels <3.5 µg/dL and ≥3.5 µg/dL in the Sample Born Between 2000 and 2009 **eTable 7.** Association Between 1-Unit (µg/dL) Increase in Lead Levels and Math and Reading Scores Within Lead Levels <3.5 µg/dL and ≥3.5 µg/dL Adding School District Specific Time Trends as Covariates **eTable 8.** Association Between 1-Unit (µg/dL) Increase in Lead Levels and Math and Reading Scores Within Lead Levels <3.5 µg/dL and ≥3.5 µg/dL Excluding Birthweight and Maternal Smoking as Covariates **eTable 9.** Association Between 1-Unit (µg/dL) Increase in Lead Levels and Math and Reading Scores Within Lead Levels <3.5 µg/dL and ≥3.5 µg/dL Separately for Males and Females

Supplement 2.
Data Sharing Statement
