# Supplementary material for: Early-Life Low Lead Levels and Academic Achievement in Childhood and Adolescence
Source: JAMA Netw Open. 2025 May 28;8(5):e2512796. doi: 10.1001/jamanetworkopen.2025.12796 (PMC12120651; doi:10.1001/jamanetworkopen.2025.12796)
Supplement: Supplement 1. — eFigure 1. Sample Construction eFigure 2. Matching of Birth Certificate and School Test Data Rates by Birth Year eFigure 3. Percent of Children with Matched Birth Certificates and School Test Data Also Matched With Lead Testing Data by Birth Year eTable 1. Regression Estimates of Association of a 1-Unit (µg/dL) Increase in Lead Levels and Math Scores Within Lead Levels <3.5 µg/dL by Grade eTable 2. Regression Estimates of Association of a 1-Unit (µg/dL) Increase in Lead Levels and Reading Scores Within Lead Levels <3.5 µg/dL by Grade eTable 3. Regression Estimates of Association of a 1-Unit (µg/dL) Increase in Lead Levels and Math Scores Within Lead Levels ≥3.5 µg/dL by Grade eTable 4. Regression Estimates of Association of a 1-Unit (µg/dL) Increase in Lead Levels and Reading Scores Within Lead Levels ≥3.5 µg/dL by Grade eTable 5. Association Between 1-Unit (µg/dL) Increase in Lead Levels and Math and Reading Scores Within Lead Levels <3.5 µg/dL and ≥3.5 µg/dL Excluding 5 µg/dL Values From Lead Mean Calculation eTable 6. Association Between 1-Unit (µg/dL) Increase in Lead Levels and Math and Reading Scores Within Lead Levels <3.5 µg/dL and ≥3.5 µg/dL in the Sample Born Between 2000 and 2009 eTable 7. Association Between 1-Unit (µg/dL) Increase in Lead Levels and Math and Reading Scores Within Lead Levels <3.5 µg/dL and ≥3.5 µg/dL Adding School District Specific Time Trends as Covariates eTable 8. Association Between 1-Unit (µg/dL) Increase in Lead Levels and Math and Reading Scores Within Lead Levels <3.5 µg/dL and ≥3.5 µg/dL Excluding Birthweight and Maternal Smoking as Covariates eTable 9. Association Between 1-Unit (µg/dL) Increase in Lead Levels and Math and Reading Scores Within Lead Levels <3.5 µg/dL and ≥3.5 µg/dL Separately for Males and Females [file jamanetwopen-e2512796-s001.pdf]

Supplementary Online Content

Wehby GL. Early life low lead level and academic achievement in childhood and adolescence. *JAMA Netw Open*. 2025;8(5):e2512796. doi:10.1001/jamanetworkopen.2025.12796.

eFigure 1. Sample Construction

eFigure 2. Matching of Birth Certificate and School Test Data Rates by Birth Year

eFigure 3. Percent of Children with Matched Birth Certificates and School Test Data Also Matched With Lead Testing Data by Birth Year

eTable 1. Regression Estimates of Association of a 1-unit (µg/dL) Increase in Lead Levels and Math Scores Within Lead Levels <3.5 µg/dL by Grade

eTable 2. Regression Estimates of Association of a 1-unit (µg/dL) Increase in Lead Levels and Reading Scores Within Lead Levels <3.5 µg/dL by Grade

eTable 3. Regression Estimates of Association of a 1-unit (µg/dL) Increase in Lead Levels and Math Scores Within Lead Levels ≥3.5 µg/dL by Grade

eTable 4. Regression Estimates of Association of a 1-unit (µg/dL) Increase in Lead Levels and Reading Scores Within Lead Levels ≥3.5 µg/dL by Grade

eTable 5. Association Between 1-unit (µg/dL) Increase in Lead Levels and Math and Reading Scores Within Lead Levels <3.5 µg/dL and ≥3.5 µg/dL Excluding 5 µg/dL Values From Lead Mean Calculation

eTable 6. Association Between 1-unit (µg/dL) Increase in Lead Levels and Math and Reading Scores Within Lead Levels <3.5 µg/dL and ≥3.5 µg/dL in the Sample Born Between 2000 and 2009

eTable 7. Association Between 1-unit (µg/dL) Increase in Lead Levels and Math and Reading Scores Within Lead Levels <3.5 µg/dL and ≥3.5 µg/dL Adding School District Specific Time Trends as Covariates

eTable 8. Association Between 1-unit (µg/dL) Increase in Lead Levels and Math and Reading Scores Within Lead Levels <3.5 µg/dL and ≥3.5 µg/dL Excluding Birthweight and Maternal Smoking as Covariates

© 2025 Wehby GL. *JAMA Network Open*.

eTable 9. Association Between 1-unit (µg/dL) Increase in Lead Levels and Math and Reading Scores Within Lead Levels <3.5 µg/dL and ≥3.5 µg/dL Separately for Males and Females

This supplementary material has been provided by the authors to give readers additional information about their work.

© 2025 Wehby GL. *JAMA Network Open*.

eFigure 1. Sample Construction

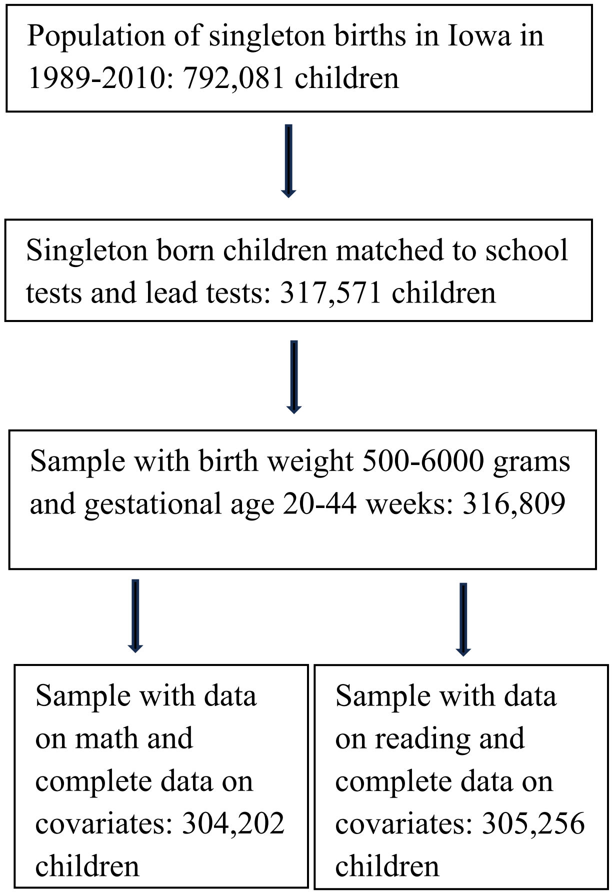

Note: The graph shows the sample construction flow.

© 2025 Wehby GL. *JAMA Network Open*.

eFigure 2. Matching of Birth Certificate and School Test Data Rates by Birth Year

Note: The rates represent the percent of singleton born children in Iowa n 1989-2010 who were matched to school test scores by birth year.

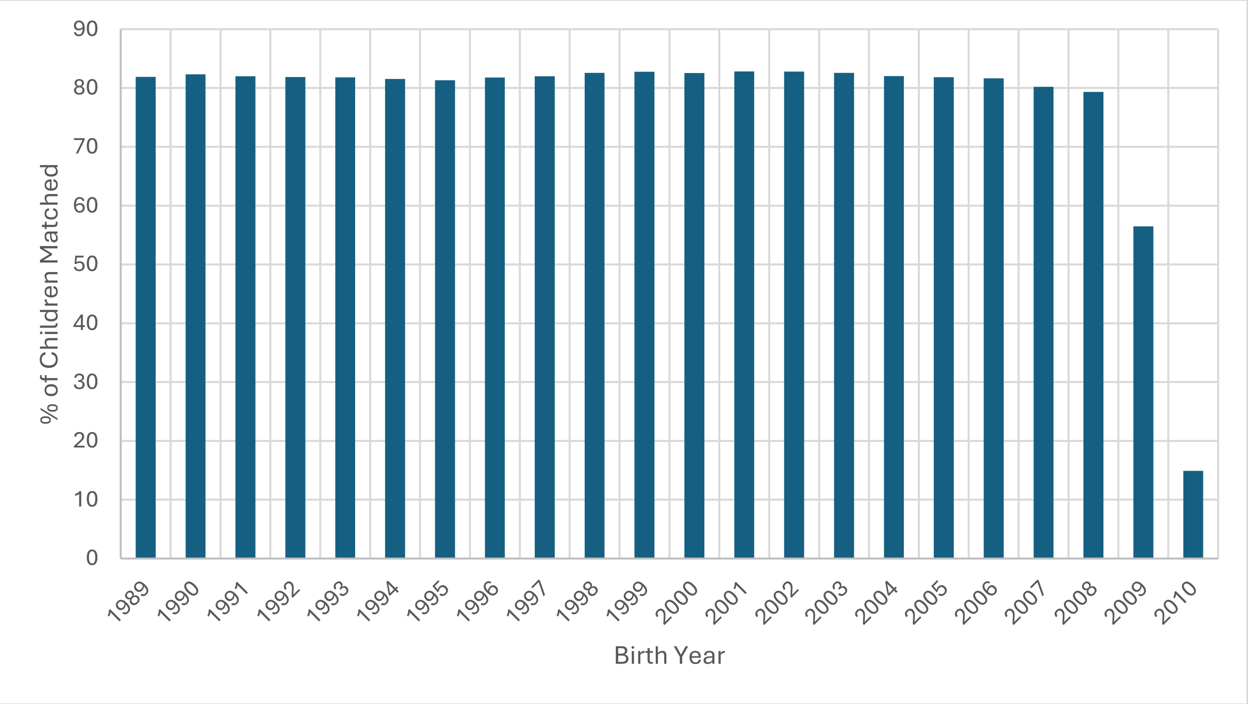

© 2025 Wehby GL. *JAMA Network Open*.

eFigure 3. Percent of Children with Matched Birth Certificates and School Test Data Also Matched With Lead Testing Data by Birth Year

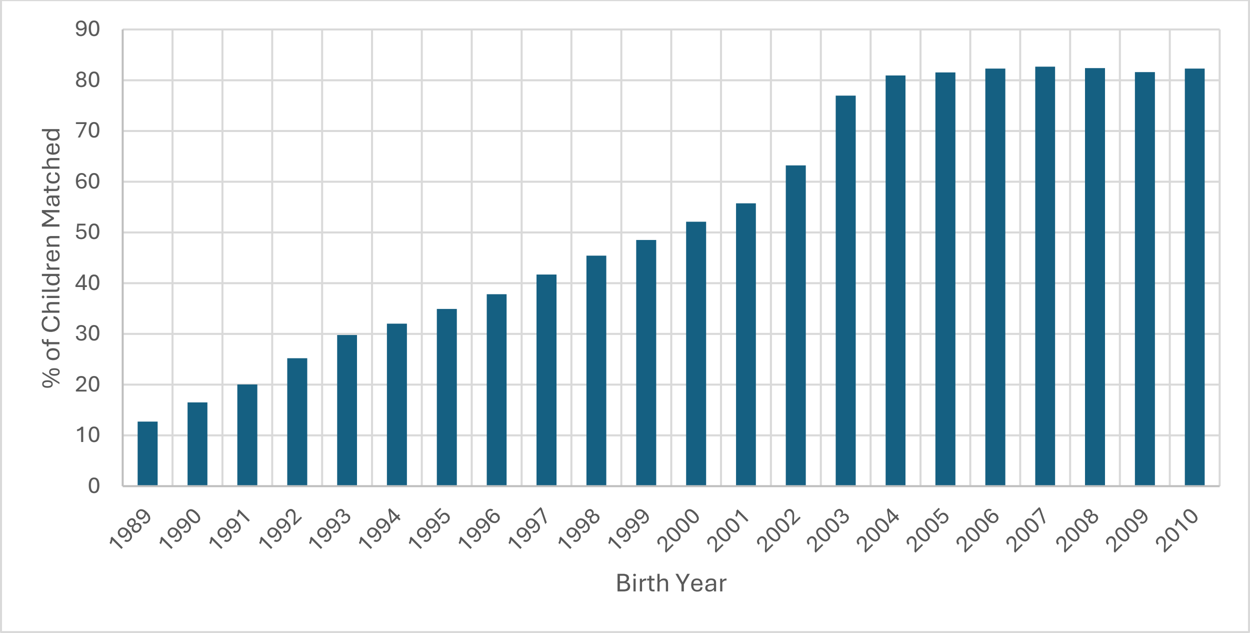

Note: The rates represent the percent of the sample of singleton born children in Iowa in 1989-2010 with matched birth certificates and school test scores who were also matched to lead tests by birth year.

© 2025 Wehby GL. *JAMA Network Open*.

eTable 1. Regression Estimates of Association of a 1-unit (μg/dL) Increase in Lead Levels and Math Scores Within Lead Levels <3.5 μg/dL by Grade

Grade	2	3	4	5	6	7	8	9	10	11
Effect of 1-unit (μg/dL) increase	-0.40**	-0.43***	-0.36***	-0.52***	-0.50***	-0.59***	-0.51***	-0.71***	-0.68***	-0.51*
95% CI	[-0.70,-0.10]	[-0.61,-0.24]	[-0.57,-0.16]	[-0.74,-0.29]	[-0.75,-0.26]	[-0.84,-0.34]	[-0.81,-0.21]	[-1.05,-0.37]	[-1.08,-0.28]	[-0.94,-0.093]
N	52317	105495	91614	77125	64397	52935	42322	32051	24171	20020

Notes: * $p < 0.05$, ** $p < 0.01$, *** $p < 0.001$; school test scores are in national percentile rankings; *N*=Number of child-grade observations. The estimates represent the association of a 1 unit-increase in lead levels with math scores estimated separately for each grade. The regression controls for child's month and year of birth, sex, gestational age (0/1 indicators for each gestational age in weeks), birth weight (0/1 indicators for birthweight deciles), 0/1 indicator for congenital anomalies, and 0/1 indicators for first year of lead testing; mother's age (0/1 indicators for each age in years), marital status, education, race, ethnicity, and number of prior live births (at child's birth), smoking, number of prenatal visits, 0/1 indicator for C-section delivery, 0/1 indicator for labor induction, and 0/1 indicator for perinatal complications; and 0/1 indicators for school districts, and year, grade (when pooling by grade), and semester at the time of school test.

© 2025 Wehby GL. *JAMA Network Open*.

eTable 2. Regression Estimates of Association of a 1-unit (µg/dL) Increase in Lead Levels and Reading Scores Within Lead Levels <3.5 µg/dL by Grade

Grade	2	3	4	5	6	7	8	9	10	11
Effect of 1-unit (µg/dL) increase	-0.62***	-0.41***	-0.28*	-0.43***	-0.41**	-0.41**	-0.34*	-0.35*	-0.36	-0.39
95% CI	[-0.92,-0.33]	[-0.61,-0.21]	[-0.50,-0.065]	[-0.66,-0.19]	[-0.67,-0.15]	[-0.70,-0.13]	[-0.66,-0.028]	[-0.68,-0.015]	[-0.74,0.025]	[-0.82,0.029]
N	52604	106076	91644	76985	64386	52986	42355	32063	24175	20106

Notes: * $p < 0.05$, ** $p < 0.01$, *** $p < 0.001$; school test scores are in national percentile rankings; N=Number of child-grade observations. The estimates represent the association of a 1 unit-increase in lead levels with reading scores estimated separately for each grade. The regression controls for child's month and year of birth, sex, gestational age (0/1 indicators for each gestational age in weeks), birth weight (0/1 indicators for birthweight deciles), 0/1 indicator for congenital anomalies, and 0/1 indicators for first year of lead testing; mother's age (0/1 indicators for each age in years), marital status, education, race, ethnicity, and number of prior live births (at child's birth), smoking, number of prenatal visits, 0/1 indicator for C-section delivery, 0/1 indicator for labor induction, and 0/1 indicator for perinatal complications; and 0/1 indicators for school districts, and year, grade (when pooling by grade), and semester at the time of school test.

© 2025 Wehby GL. *JAMA Network Open*.

eTable 3. Regression Estimates of Association of a 1-unit (µg/dL) Increase in Lead Levels and Math Scores Within Lead Levels ≥3.5 µg/dL by Grade

Grade	2	3	4	5	6	7	8	9	10	11
Effect of 1-unit (µg/dL) increase	-0.50***	-0.52***	-0.52***	-0.51***	-0.55***	-0.57***	-0.53***	-0.54***	-0.50***	-0.45***
95% CI	[-0.60,-0.40]	[-0.59,-0.46]	[-0.59,-0.45]	[-0.58,-0.44]	[-0.62,-0.48]	[-0.64,-0.51]	[-0.60,-0.46]	[-0.61,-0.47]	[-0.57,-0.42]	[-0.53,-0.38]
N	82786	171719	163416	151575	140102	128106	116243	98901	83507	76697

Notes: * $p < 0.05$, ** $p < 0.01$, *** $p < 0.001$; school test scores are in national percentile rankings; N=Number of child-grade observations. The estimates represent the association of a 1 unit-increase in lead levels with math scores estimated separately for each grade. The regression controls for child's month and year of birth, sex, gestational age (0/1 indicators for each gestational age in weeks), birth weight (0/1 indicators for birthweight deciles), 0/1 indicator for congenital anomalies, and 0/1 indicators for first year of lead testing; mother's age (0/1 indicators for each age in years), marital status, education, race, ethnicity, and number of prior live births (at child's birth), smoking, number of prenatal visits, 0/1 indicator for C-section delivery, 0/1 indicator for labor induction, and 0/1 indicator for perinatal complications; and 0/1 indicators for school districts, and year, grade (when pooling by grade), and semester at the time of school test.

© 2025 Wehby GL. *JAMA Network Open*.

eTable 4. Regression Estimates of Association of a 1-unit (µg/dL) Increase in Lead Levels and Reading Scores Within Lead Levels ≥3.5 µg/dL by Grade

Grade	2	3	4	5	6	7	8	9	10	11
Effect of 1-unit (µg/dL) increase	-0.65***	-0.55***	-0.55***	-0.53***	-0.59***	-0.60***	-0.62***	-0.56***	-0.54***	-0.47***
95% CI	[-0.74,-0.55]	[-0.62,-0.48]	[-0.62,-0.48]	[-0.60,-0.46]	[-0.66,-0.51]	[-0.67,-0.53]	[-0.69,-0.55]	[-0.63,-0.49]	[-0.62,-0.47]	[-0.54,-0.39]
N	87517	172888	163540	151434	140186	128217	116393	98951	83582	76785

Notes: * $p < 0.05$, ** $p < 0.01$, *** $p < 0.001$; school test scores are in national percentile rankings; *N*=Number of child-grade observations. The estimates represent the association of a 1 unit-increase in lead levels with reading scores estimated separately for each grade. The regression controls for child's month and year of birth, sex, gestational age (0/1 indicators for each gestational age in weeks), birth weight (0/1 indicators for birthweight deciles), 0/1 indicator for congenital anomalies, and 0/1 indicators for first year of lead testing; mother's age (0/1 indicators for each age in years), marital status, education, race, ethnicity, and number of prior live births (at child's birth), smoking, number of prenatal visits, 0/1 indicator for C-section delivery, 0/1 indicator for labor induction, and 0/1 indicator for perinatal complications; and 0/1 indicators for school districts, and year, grade (when pooling by grade), and semester at the time of school test.

© 2025 Wehby GL. *JAMA Network Open*.

eTable 5. Association Between 1-unit (μg/dL) Increase in Lead Levels and Math and Reading Scores Within Lead Levels <3.5 μg/dL and ≥3.5 μg/dL Excluding 5 μg/dL Values From Lead Mean Calculation

	Lead levels <3.5 μg/dL		Lead levels ≥3.5 μg/dL	
	NPR_MT	NPR_RC	NPR_MT	NPR_RC
Estimate	-0.37***	-0.30***	-0.31***	-0.31***
95% CI	[-0.55,-0.20]	[-0.48,-0.12]	[-0.37,-0.25]	[-0.37,-0.25]
N	566715	567634	407266	409447

Notes: * $p < 0.05$, ** $p < 0.01$, *** $p < 0.001$; school test scores are in national percentile rankings; *N*=Number of child-grade observations. Notes: * $p < 0.05$, ** $p < 0.01$, *** $p < 0.001$; school test scores are in national percentile rankings; *N*=Number of child-grade observations. The estimates represent the association of a 1 unit-increase in lead levels with math and reading scores. The regression controls for child's month and year of birth, sex, gestational age (0/1 indicators for each gestational age in weeks), birth weight (0/1 indicators for birthweight deciles), 0/1 indicator for congenital anomalies, and 0/1 indicators for first year of lead testing; mother's age (0/1 indicators for each age in years), marital status, education, race, ethnicity, and number of prior live births (at child's birth), smoking, number of prenatal visits, 0/1 indicator for C-section delivery, 0/1 indicator for labor induction, and 0/1 indicator for perinatal complications; and 0/1 indicators for school districts, and year, grade (when pooling by grade), and semester at the time of school test.

© 2025 Wehby GL. *JAMA Network Open*.

eTable 6. Association Between 1-unit (µg/dL) Increase in Lead Levels and Math and Reading Scores Within Lead Levels <3.5 µg/dL and ≥3.5 µg/dL in the Sample Born Between 2000 and 2009

	Lead levels <3.5 µg/dL		Lead levels ≥3.5 µg/dL	
	NPR_MT	NPR_RC	NPR_MT	NPR_RC
Estimate	-0.40***	-0.36***	-0.52***	-0.59***
95% CI	[-0.60,-0.21]	[-0.47,-0.26]	[-0.62,-0.42]	[-0.70,-0.49]
N	419771	229614	599572	601770

Notes: * $p < 0.05$, ** $p < 0.01$, *** $p < 0.001$; school test scores are in national percentile rankings; *N*=Number of child-grade observations. The estimates represent the association of a 1 unit-increase in lead levels with math and reading scores. The regression controls for child's month and year of birth, sex, gestational age (0/1 indicators for each gestational age in weeks), birth weight (0/1 indicators for birthweight deciles), 0/1 indicator for congenital anomalies, and 0/1 indicators for first year of lead testing; mother's age (0/1 indicators for each age in years), marital status, education, race, ethnicity, and number of prior live births (at child's birth), smoking, number of prenatal visits, 0/1 indicator for C-section delivery, 0/1 indicator for labor induction, and 0/1 indicator for perinatal complications; and 0/1 indicators for school districts, and year, grade (when pooling by grade), and semester at the time of school test.

© 2025 Wehby GL. *JAMA Network Open*.

eTable 7. Association Between 1-unit (µg/dL) Increase in Lead Levels and Math and Reading Scores Within Lead Levels <3.5 µg/dL and ≥3.5 µg/dL Adding School District Specific Time Trends as Covariates

	Lead levels <3.5 µg/dL		Lead levels ≥3.5 µg/dL	
	NPR_MT	NPR_RC	NPR_MT	NPR_RC
Estimate	-0.48***	-0.43***	-0.52***	-0.56***
95% CI	[-0.67,-0.30]	[-0.62,-0.24]	[-0.57,-0.46]	[-0.61,-0.50]
N	562447	563380	1213052	1219493

Notes: * p < 0.05, ** p < 0.01, *** p < 0.001; school test scores are in national percentile rankings; *N*=Number of child-grade observations. Notes: * p < 0.05, ** p < 0.01, *** p < 0.001; school test scores are in national percentile rankings; *N*=Number of child-grade observations. The estimates represent the association of a 1 unit-increase in lead levels with math and reading scores. The regression controls for child's month and year of birth, sex, gestational age (0/1 indicators for each gestational age in weeks), birth weight (0/1 indicators for birthweight deciles), 0/1 indicator for congenital anomalies, and 0/1 indicators for first year of lead testing; mother's age (0/1 indicators for each age in years), marital status, education, race, ethnicity, and number of prior live births (at child's birth), smoking, number of prenatal visits, 0/1 indicator for C-section delivery, 0/1 indicator for labor induction, and 0/1 indicator for perinatal complications; and 0/1 indicators for school districts, and year, grade (when pooling by grade), and semester at the time of school test. The regression also controls for school district specific time trends (added as interactions between school district and year of birth fixed effects).

© 2025 Wehby GL. *JAMA Network Open*.

eTable 8. Association Between 1-unit (µg/dL) Increase in Lead Levels and Math and Reading Scores Within Lead Levels <3.5 µg/dL and ≥3.5 µg/dL Excluding Birthweight and Maternal Smoking as Covariates

	Lead levels <3.5 µg/dL		Lead levels ≥3.5 µg/dL	
	NPR_MT	NPR_RC	NPR_MT	NPR_RC
Estimate	-0.50***	-0.41***	-0.56***	-0.59***
95% CI	[-0.68,-0.32]	[-0.59,-0.22]	[-0.61,-0.50]	[-0.65,-0.54]
N	562648	563581	1213338	1219781

Notes: * $p < 0.05$, ** $p < 0.01$, *** $p < 0.001$; school test scores are in national percentile rankings; *N*=Number of child-grade observations. Notes: * $p < 0.05$, ** $p < 0.01$, *** $p < 0.001$; school test scores are in national percentile rankings; *N*=Number of child-grade observations. The estimates represent the association of a 1 unit-increase in lead levels with math and reading scores. The regression controls for child's month and year of birth, sex, gestational age (0/1 indicators for each gestational age in weeks), 0/1 indicator for congenital anomalies, and 0/1 indicators for first year of lead testing; mother's age (0/1 indicators for each age in years), marital status, education, race, ethnicity, and number of prior live births (at child's birth), number of prenatal visits, 0/1 indicator for C-section delivery, 0/1 indicator for labor induction, and 0/1 indicator for perinatal complications; and 0/1 indicators for school districts, and year, grade (when pooling by grade), and semester at the time of school test. The regression also controls for school district specific time trends (added as interactions between school district and year of birth fixed effects).

© 2025 Wehby GL. *JAMA Network Open*.

eTable 9. Association Between 1-unit (µg/dL) Increase in Lead Levels and Math and Reading Scores Within Lead Levels <3.5 µg/dL and ≥3.5 µg/dL Separately for Males and Females

	Lead levels <3.5 µg/dL		Lead levels ≥3.5 µg/dL	
	NPR_MT	NPR_RC	NPR_MT	NPR_RC
Males				
Estimate	-0.40**	-0.32*	-0.53***	-0.55***
95% CI	[-0.66,-0.15]	[-0.58,-0.055]	[-0.61,-0.45]	[-0.62,-0.47]
N	284122	284534	620818	624043
Females				
Estimate	-0.55***	-0.45***	-0.50***	-0.58***
95% CI	[-0.79,-0.30]	[-0.70,-0.21]	[-0.58,-0.43]	[-0.66,-0.50]
N	278325	278846	592234	595450

Notes: * p < 0.05, ** p < 0.01, *** p < 0.001; school test scores are in national percentile rankings; *N*=Number of child-grade observations. Notes: * p < 0.05, ** p < 0.01, *** p < 0.001; school test scores are in national percentile rankings; *N*=Number of child-grade observations. The estimates represent the association of a 1 unit-increase in lead levels with math and reading scores. The regression controls for child's month and year of birth, gestational age (0/1 indicators for each gestational age in weeks), 0/1 indicator for congenital anomalies, and 0/1 indicators for first year of lead testing; mother's age (0/1 indicators for each age in years), marital status, education, race, ethnicity, and number of prior live births (at child's birth), number of prenatal visits, 0/1 indicator for C-section delivery, 0/1 indicator for labor induction, and 0/1 indicator for perinatal complications; and 0/1 indicators for school districts, and year, grade (when pooling by grade), and semester at the time of school test. The regression also controls for school district specific time trends (added as interactions between school district and year of birth fixed effects).

© 2025 Wehby GL. *JAMA Network Open*.
