# Supplementary material for: Early-Life Low Lead Levels and Academic Achievement in Childhood and Adolescence
Source: JAMA Netw Open. 2025 May 28;8(5):e2512796. doi: 10.1001/jamanetworkopen.2025.12796 (PMC12120651; doi:10.1001/jamanetworkopen.2025.12796)
Supplement: Supplement 2. — Data Sharing Statement [file jamanetwopen-e2512796-s002.pdf]

# Data Sharing Statement

Wehby. Early-Life Low Lead Levels and Academic Achievement in Childhood and Adolescence. *JAMA Netw Open*. Published May 28, 2025.
doi:10.1001/jamanetworkopen.2025.12796

**Data**
**Data available:** No

**Additional Information**
**Explanation for why data not available:** The data uses restricted access datasets governed by data use agreements that do no allow the researcher to release the data publicly. Interested researchers should contact the data providing agencies to access the data.
